# Relation among EGFL7, ITGB3, and KLF2 and their clinical implication in multiple myeloma patients: a prospective study

**DOI:** 10.1007/s11845-021-02781-2

**Published:** 2021-10-11

**Authors:** Yaqiong Li, Lingli Zhang, Jichang Gong

**Affiliations:** Department of Hematology, Dazhou Central Hospital, Tongchuan District, 56 Nanyue Temple Street Sichuan, Dazhou, 635000 China

**Keywords:** EGF like-7, Integrin subunit beta 3, Kruppel-like factor 2, Multiple myeloma, Prognosis

## Abstract

**Objective:**

We aimed to investigate the relationship among epidermal growth factor–like protein-7 (EGFL7), integrin subunit beta 3 (ITGB3), and Kruppel-like factor 2 (KLF2) expressions and their clinical implication in multiple myeloma (MM).

**Methods:**

This prospective study enrolled 72 de novo symptomatic MM patients and 30 controls, and then collected their bone marrow plasma cell samples. Subsequently, the EGFL7, ITGB3, and KLF2 expressions were carried out by reverse transcription quantitative polymerase chain reaction.

**Results:**

EGFL7, ITGB3, and KLF2 expressions were increased in MM patients compared to controls. Besides, EGFL7, ITGB3, and KLF2 inter-correlated with each other in MM patients but not in controls. In MM patients, EGFL7 and ITGB3 (but not KLF2) expressions were positively correlated with ISS stage, while ITGB3 and KLF2 (but not EGFL7) expressions were correlated with increased R-ISS stage. Interestingly, ITGB3 and KLF2 were decreased in induction-treatment complete remission (CR) MM patients compared to non-CR MM patients, while EGFL7 only showed a trend but without statistical significance. Furthermore, ITGB3 high expression was correlated with worse progression-free survival (PFS) and overall survival (OS), while EGFL7 and KLF2 high expressions only associated with pejorative PFS but not OS.

**Conclusion:**

EGFL7, ITGB3, and KLF2 may serve as potential prognostic indicators in MM patients.

**Supplementary Information:**

The online version contains supplementary material available at 10.1007/s11845-021-02781-2.

## Introduction

Multiple myeloma (MM) is a malignancy pathologically characterized by a neoplastic plasma cell disorder with proliferation of clonal plasma cells in the bone marrow and monoclonal protein in the blood or urine [1, 2]. MM is reported as the second most common hematologic malignancies (occupying nearly 1% of neoplastic diseases and 13% of hematologic malignancies), which represents the high incidence and mortality rate with the estimated new cases of 176,404 and deaths of 117,077 worldwide in 2020; besides in China, it exhibits a rough prevalence and incidence of 6.88 per 100,000 populations and 1.60 per 100,000 populations annually, respectively [1, 3]. Besides, the MM also represents the high incidence in the world. In the recent years, the diagnosis and treatment of MM have continuously improved, which are related to improved prognosis in MM patients. However, MM is still incurable. Thus, identifying potential biomarkers may have a good clinical meaning for the improvement of MM prognosis.

The angiogenic factor (angiogenesis-promoting factor) epidermal growth factor like protein-7 (EGFL7) has emerged as a chemoattractant for endothelial cells and a key factor regulating vascular development [4]. Recently, EGFL7 has dysregulated expression in various solid cancer patients (such as cholangiocarcinoma [5], hepatocellular carcinoma [6], and osteosarcoma [7]). Excepting solid cancers, there are also several reports involving in the role of EGFL7 in hematologic malignancy patients. For instance, EGFL7 is highly expressed, and its high expression associates with lower complete remission rates in acute myeloid leukemia (AML) patients [8]. Regarding MM, the EGFL7 has been reported to interact with integrin subunit beta 3 (ITGB3) and Kruppel-like factor 2 (KLF2) to enhance cell adhesion and promote cell survival in MM [9]. Besides, the ITGB3 and KLF2 have also been reported to play a tumorigenesis role in the pathogenesis of MM in vitro [9, 10]. No report could be found involving in the clinical implication of EGFL7, ITGB3, and KLF2 in MM patients. In the present study, we aimed to investigate the relationship among EGFL7, ITGB3, KLF2 and their association with clinical features, treatment response, and survival profiles in MM patients.

## Methods

### Subjects

After being approved by Institutional Review Board, this prospective study consecutively enrolled 72 de novo symptomatic MM patients between January 2017 and June 2020. The patients were required to meet the following inclusion criteria: (1) newly diagnosed as symptomatic MM in accordance with National Comprehensive Cancer Network (NCCN) Guideline (Version 3.2016) [11], (2) age more than 18 years, (3) volunteer to participate in the present study and agreed with the collection of bone marrow (BM) sample for study use, (4) able to complete study follow-up. The following patients were ineligible for inclusion: (1) secondary MM or relapsed MM; (2) smoldering MM; (3) presented with other malignant diseases; (4) received radiotherapy or chemotherapy before enrollment; (5) gestational patients. Additionally, this study also recruited 30 healthy BM donors as health controls. All subject signed informed consents before recruitment.

### Baseline data collection

Demographics, clinical manifestations, chromosomal abnormalities, and disease stage of patients were recorded after diagnostic examinations, among which staging for MM patients was conducted referring to Durie-Salmon staging system, International Staging System (ISS), and revised ISS (R-ISS) [12–14].

### Sample collection and RT-qPCR

BM samples were respectively acquired from MM patients at diagnosis and from health donors at donation, which were then submitted to isolate plasma cells using CD138-immunomagnetic beads (cat. no. 130–051-301, Miltenyi Biotec, Paris, France) in accordance with product manual. Subsequently, quantitative analysis of EGFL7, ITGB3, and KLF2 expression was carried out by reverse transcription quantitative polymerase chain reaction (RT-qPCR). The detailed processes of RT-qPCR were as follows: (1) total RNA was extracted using RNeasy Protect Mini Kit (cat. no. 74124, Qiagen, Duesseldorf, Nordrhein-Westfalen, Germany). (2) The RNA was conversed to cDNA using ReverTra Ace® qPCR RT Kit (cat. no. FSQ-201, Toyobo, Osaka, Kansai, Japan). (3) The quantitative qPCR was carried out using KOD SYBR® qPCR Mix (cat. no. 7772383, Toyobo, Osaka, Kansai, Japan). (4) The PCR amplification was conducted as follows: 95 °C for 3 min, 40 cycles of 95 °C for 5 s, 61 °C for 10 s, 72 °C for 30 s. Glyceraldehyde-3-phosphate dehydrogenase (GAPDH) was served as the internal reference. The calculation of mRNA expressions was according to 2^−ΔΔCt^ formula [15]. Primer design was conducted with a previous study used as reference [9].

### Response and survival assessment

All patients received the regimen of lenalidomide/bortezomib/dexamethasone as induction treatment. Response to induction therapy was assessed at week 6 after the induction therapy referring to International Myeloma Working Group (IMWG) criteria [16]. As for survival analysis, regular follow-up (every 3 months or as clinically indicated) was conducted until 2021 Jan. 31. Progression-free survival (PFS) and overall survival (OS) were assessed according to the IMWG guideline [16]. Patients who died during induction therapy or did not undergo the assessment of response to induction therapy due to early loss of follow-up had been excluded from the study analysis.

### Statistical analysis

Characteristics of continuous variables were described as mean with standard deviation (SD) or median with interquartile range (IQR) depending on the normality determined by the Kolmogorov–Smirnov (K-S) test. Categorical variables were described as number and percentage. Expression difference of genes between groups was analyzed by Mann–Whitney *U* test. Correlation of genes with clinical features of MM patients was analyzed using the Spearman test or Kruskal–Wallis *H* rank sum test. The performance of genes in discriminating different subjects was estimated by receiver operating characteristic (ROC) curve and the area under the curve (AUC). Survival difference between patients with different expression of genes was compared by log-rank test, which was shown in Kaplan–Meier curves. It is of note that for survival analysis, patients were categorized into cases with high gene (EGFL7, ITGB3, and KLF2) expression and cases with low gene (EGFL7, ITGB3, and KLF2) expression according to the median gene expression in MM patients, respectively. The independent factors influencing the PFS and OS were determined by the multivariate Cox’s proportional hazards regression analysis. Statistical significance was defined as *P* value < 0.05 in the analysis. SPSS 24.0 (IBM Corp., Armonk, New York, USA) was used for data analysis.

## Results

### MM patients’ clinical features

In 72 MM patients (including 42 (58.3%) males and 30 (41.7%) females), the mean age was 54.5 ± 8.2 years. The detailed information about immunoglobulin subtype, bone lesion, renal impairment, biochemical indexes, chromosomal abnormality, Durie-Salmon stage, ISS stage, and R-ISS stage is shown in Table 1.

**Table 1:** Clinical features of MM patients

Items	MM patients (*N* = 72)
Age (years), mean ± SD	54.5 ± 8.2
Gender, No. (%)	
Male	42 (58.3)
Female	30 (41.7)
Immunoglobulin subtype, no. (%)	
IgG	40 (55.5)
IgA	13 (18.1)
Others	19 (26.4)
Bone lesion, no. (%)	
Yes	55 (76.4)
No	17 (23.6)
Renal impairment, no. (%)	
Yes	33 (45.8)
No	39 (54.2)
Biochemical indexes	
Hb (g/L), mean ± SD	98.8 ± 25.4
Calcium (mg/dL), mean ± SD	9.9 ± 2.0
Scr (mg/dL), median (IQR)	1.9 (1.3–2.4)
ALB (g/L), median (IQR)	33.0 (27.3–37.0)
β_2_-MG (mg/L), median (IQR)	5.8 (3.0–9.8)
LDH (U/L), median (IQR)	204.1 (172.8–250.4)
Chromosomal abnormality, no. (%)	
*t* (4; 14)	7 (9.7)
*t* (14; 16)	3 (4.2)
Del (17p)	6 (8.3)
Durie-Salmon stage, no. (%)	
I	0 (0.0)
II	6 (8.3)
III	66 (91.7)
ISS stage, no. (%)	
I	7 (9.7)
II	26 (36.1)
III	39 (54.2)
R-ISS stage, no. (%)	
I	4 (5.6)
II	35 (48.6)
III	33 (45.8)

*MM* multiple myeloma, *SD* standard deviation, *IgG* immunoglobulin G, *IgA* immunoglobulin A, *Hb* hemoglobin, *Scr* serum creatinine, *IQR* interquartile range, *ALB* albumin, *β*_*2*_*-MG* Beta-2-microglobulin, *LDH* lactate dehydrogenase, *ISS* International Staging System, *R-ISS* revised International Staging System

### EGFL7, ITGB3, and KLF2 expressions

**Fig. 1:**
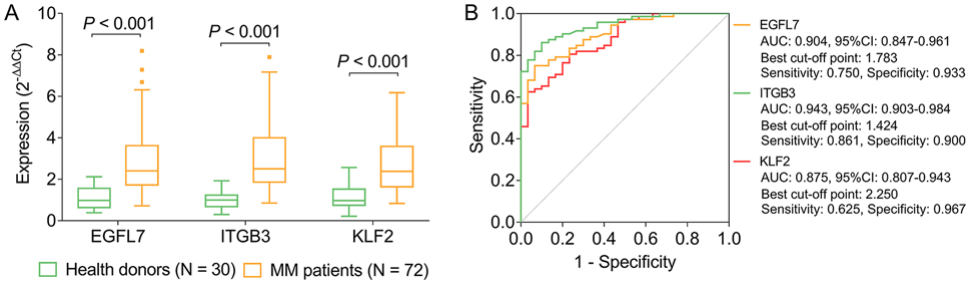
The expressions of EGFL7, ITGB3, and KLF2 in MM patients and health donors. Comparison of EGFL7, ITGB3, and KLF2 expressions between MM patients and health donors (**A**). ROC curves of EGFL7, ITGB3, and KLF2 for MM risk (**B**). EGFL7 epidermal growth factor like protein-7; ITGB3 integrin subunit beta 3; KLF2 Kruppel-like factor 2; MM multiple myeloma; ROC receiver operating characteristic; AUC area under the curve; CI confidence interval

EGFL7 (*P* < 0.001), ITGB3 (*P* < 0.001), and KLF2 (*P* < 0.001) expressions were increased in MM patients compared to health donors (Fig. 1A). Further ROC curves showed that EGFL7 (AUC 0.904, 95% CI (0.847–0.961)), ITGB3 (AUC 0.943, 95% CI (0.903–0.984)), and KLF2 (AUC 0.875, 95% CI (0.807–0.943)) could distinguish MM patients from health donors, and the sensitivity and specificity of EGFL7, ITGB3, and KLF2 were 0.750 and 0.933, 0.861 and 0.900, and 0.625 and 0.967, respectively, at the best cut-off points (Fig. 1B).

### Intercorrelation among EGFL7, ITGB3, and KLF2 expressions

**Fig. 2:**
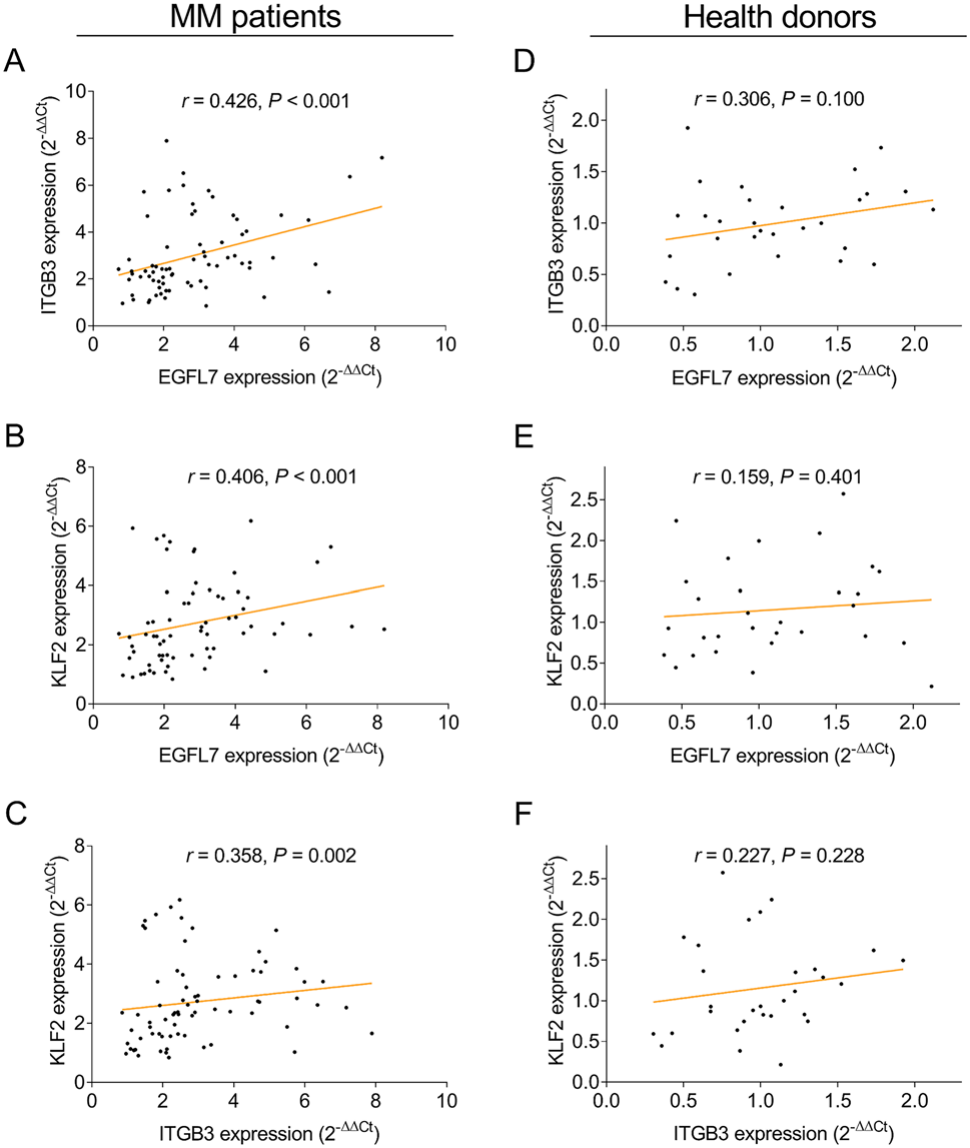
Intercorrelation among EGFL7, ITGB3, and KLF2 expressions in MM patients and health donors. Correlation of EGFL7 with ITGB3 (**A**), EGFL7 with KLF2 (**B**), and ITGB3 with KLF2 (**C**) in MM patients. Correlation of EGFL7 with ITGB3 (**D**), EGFL7 with KLF2 (**E**), and ITGB3 with KLF2 (**F**) in health donors. EGFL7 epidermal growth factor like protein-7; ITGB3 integrin subunit beta 3; KLF2 Kruppel-like factor 2; MM multiple myeloma

In MM patients, EGFL7 expression was positively correlated with ITGB3 expression (*P* < 0.001) (Fig. 2A) and KLF2 expression (*P* < 0.001) (Fig. 2B); also, ITGB3 expression was positively associated with KLF2 expression (*P* = 0.002) (Fig. 2C). In health donors, EGFL7 expression was not correlated with ITGB3 expression (*P* = 0.100) (Fig. 2D) and KLF2 expression (*P* = 0.401) (Fig. 2E); also, ITGB3 expression was not associated with KLF2 expression (*P* = 0.228) (Fig. 2F).

### Correlation of EGFL7, ITGB3, and KLF2 expressions with clinical features

**Fig. 3:**
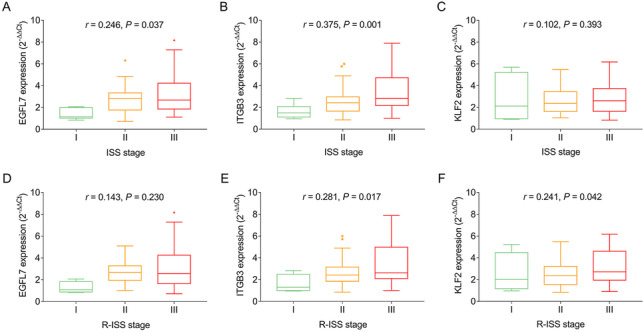
Relationship of EGFL7, ITGB3, and KLF2 expressions with prognostic risk stratifications in MM patients. Correlation of EGFL7 (**A**), ITGB3 (**B**), and KLF2 (**C**) with ISS stage. Correlation of EGFL7 (**D**), ITGB3 (**E**), and KLF2 (**F**) with R-ISS stage. EGFL7 epidermal growth factor like protein-7; ITGB3 integrin subunit beta 3; KLF2 Kruppel-like factor 2; MM multiple myeloma; ISS International Staging System; R-ISS revised International Staging System

**Fig. 4:**
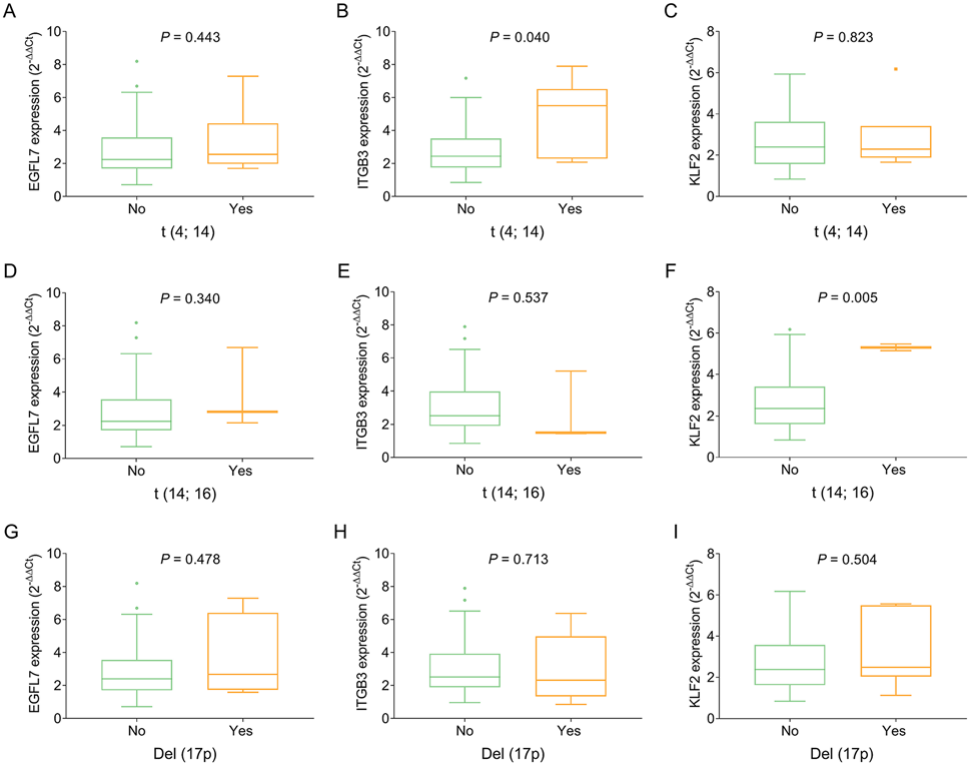
Relationship of EGFL7, ITGB3 and KLF2 expressions with chromosomal abnormality in MM patients. Correlation of EGFL7 (**A**), ITGB3 (**B**), and KLF2 (**C**) expressions with *t* (4; 14) occurrence. Correlation of EGFL7 (**D**), ITGB3 (**E**), and KLF2 (**F**) expressions with t (14; 16) occurrence. Correlation of EGFL7 (**G**), ITGB3 (**H**), and KLF2 (**I**) expression with Del (17p) occurrence. EGFL7 epidermal growth factor like protein-7; ITGB3 integrin subunit beta 3; KLF2 Kruppel-like factor 2; MM multiple myeloma

In MM patients, EGFL7 (*P* = 0.037) (Fig. 3A) and ITGB3 (*P* = 0.001) (Fig. 3B) were positively correlated with ISS stage, while no correlation of KLF2 expression with ISS stage (*P* = 0.393) (Fig. 3C) was found. In addition, ITGB3 (*P* = 0.017) (Fig. 3E) and KLF2 (*P* = 0.042) (Fig. 3F) were positively correlated with R-ISS stage, while no correlation of EGFL7 expression with R-ISS stage (*P* = 0.230) (Fig. 3D) was found. In addition, ITGB3 expression (*P* = 0.040) (Fig. 4B) was correlated with *t* (4; 14), and KLF2 expression (*P* = 0.005) (Fig. 4F) was correlated with *t* (4; 16). However, there was no correlation of EGFL7, ITGB3, and KLF2 with other chromosomal abnormalities (Fig. 4A, C–E, G–I).

Besides, the correlation of EGFL7, ITGB3, and KLF2 with immunoglobulin subtype was also determined, which is shown in Supplemental Fig. 1A-C.

### Correlation of EGFL7, ITGB3, and KLF2 expressions with treatment response

**Fig. 5:**
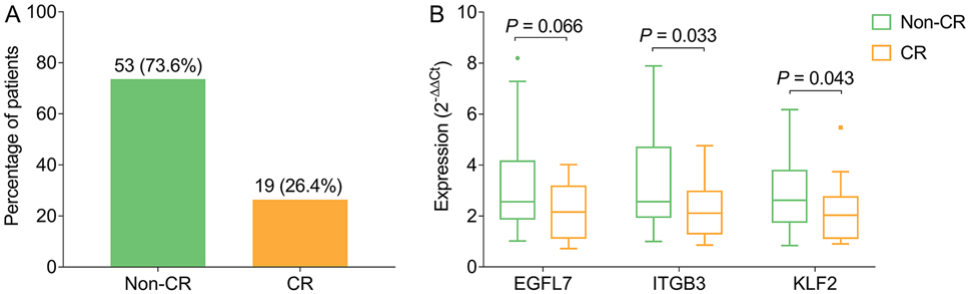
Relationship of EGFL7, ITGB3, and KLF2 expressions with treatment response in MM patients. Percentage of patients with CR and non-CR (**A**). Compression of EGFL7, ITGB3 and KLF2 between CR patients and non-CR patients (**B**). EGFL7 epidermal growth factor like protein-7; ITGB3 integrin subunit beta 3; KLF2 Kruppel-like factor 2; MM multiple myeloma; CR complete remission

There were 19 (26.4%) complete remission (CR) patients and 53 (73.6%) non-CR patients (Fig. 5A). After induction treatment, ITGB3 expression (*P* = 0.033) and KLF2 expression (*P* = 0.043), but not EGFL7 expression (*P* = 0.066), were decreased in CR patients compared to non-CR patients (Fig. 5B).

### Correlation of EGFL7, ITGB3, and KLF2 expressions with PFS and OS

**Fig. 6:**
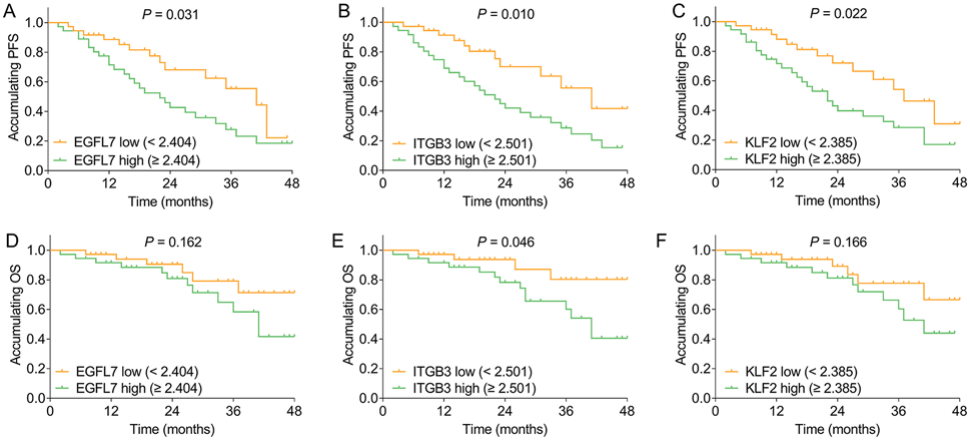
Relationship of EGFL7, ITGB3 and KLF2 expressions with survival profiles in MM patients. Correlation of EGFL7 (**A**), ITGB3 (**B**), and KLF2 (**C**) with PFS. Correlation of EGFL7 (**D**), ITGB3 (**E**), and KLF2 (**F**) with OS. EGFL7 epidermal growth factor like protein-7; ITGB3 integrin subunit beta 3; KLF2 Kruppel-like factor 2; MM multiple myeloma; PFS progression-free survival; OS overall survival

High expressions of EGFL7 (*P* = 0.031) (Fig. 6A), ITGB3 (*P* = 0.010) (Fig. 6B), and KLF2 (*P* = 0.022) (Fig. 6C) were correlated with worse PFS. In addition, high expression of ITGB3 (*P* = 0.046) (Fig. 6E), but not EGFL7 (*P* = 0.163) (Fig. 6D) or KLF2 (*P* = 0.166) (Fig. 6F) expression, was correlated with poor OS.

To determine the independent factors influencing the PFS and OS, the multivariate Cox’s proportional hazards regression analysis for PFS and OS was performed, which was shown in Supplemental Table 1.

## Discussion

Clinically, a previous study reveals that EGFL7 serves as a potential diagnostic marker in B-cell acute lymphoblastic leukemia (B-ALL) patients [17]. Another recent study displays that EGFL7 is overexpressed in AML patients compared to controls, and its overexpression relates to lower complete remission rates in AML patients [8]. These data indicate that EGFL7 plays an important role in hematologic malignancy patients, while no evidence is found involving in the clinical value of EGFL7 in MM patients. In the current study, we discovered that EGFL7 could distinguish MM patients from health donors; also, it positively correlated with increased ISS stage and worse PFS in MM patients. The possible explanations were (1) EGFL7 could regulate several pathways (including EGF receptor (EGFR)/protein kinase B (AKT) signaling) to promote cell proliferation, thereby accelerated MM progression [18]. Collectively, EGFL7 high expression was related to increased risk of MM. (2) EGFL7 could regulate extracellular signal-regulated kinase (ERK), signal transducer, and activator of transcription 3 (STAT3) and integrin signaling cascades to induce endothelial cell activities and form angiogenic regulation in the bone microenvironment, which further promoted MM progression, thereby related to worse prognostic risk stratification (ISS/R-ISS) and poor survival in MM patients [4]. Besides, we also found the intercorrelation among EGFL7, ITGB3, and KLF2, which might be explained as follows: (1) EGFL7 could interact with the ITGB3 and KLF2 to enhance cell adhesion and promote cell survival in MM; thus, they intercorrelated [9]. (2) Elevated expression of EGFL7, ITGB3, and KLF2 was reported to be associated with the reduced chemosensitivity of patients with tumors; thus, they were intercorrelated [7, 19, 20].

ITGB3 has been reported as a promoter in various carcinomas including breast cancer [21], hepatocellular carcinoma [22], and gastric cancer [23]. More importantly, ITGB3 is regarded as a reliable marker for defining hemogenic endothelial cells during hematopoiesis, which establishes a robust platform for dissecting hematopoiesis, which may cause the generation of HSCs in vitro [24]. In addition, ITGB3 interacts with STAT6 to participant in the progression of AML [25]. However, few studies have been performed for the clinical implication of ITGB3 in hematologic malignancy patients, participants in MM patients. Our study discovered that ITGB3 could distinguish MM patients from health donors; besides, it was positively related to ISS stage and R-ISS stage. Also, its high expression was correlated with decreased CR and worse PFS. OS. Three reasons might exist: (1) ITGB3 could upregulate the expression of transcription factor KLF2 (a critical regulator for MM cell survival), and thereby enhanced MM cell proliferation [10]. Hence, ITGB3 was related to high risk of MM. (2) ITGB3 might regulate several genes to subsequently affect albumin, β2-MG, or LDH, Hence, ITGB3 was related to raised ISS stage and R-ISS stage in MM patients. (3) As our results about chromosomal abnormalities, ITGB3 might be correlated with *t* (4; 14) in MM patients, and the translocation *t* (4;14) was a critical cytogenetic change of MM, which was related to worse prognostic risk stratification and a poor prognosis [26]. Hence, ITGB3 high expression was correlated with worse prognosis in MM patients. In addition, ITGB3 appears to have a stronger prognostic value, which might be associated with more prognostic risk stratification–related factors.

KLF2, a member of the zinc finger family, has been illustrated to be involved in the pathology of hematologic malignancies [27, 28]. For instance, KIF2 interacts with adenosine monophosphate–activated protein kinase (AMPK) and *Liriope muscari* baily saponins C (DT-13) to promote cell apoptosis and differentiation in AML [29]. Furthermore, KLF2 binds with lysine demethylase 3A (KDM3A) and interferon regulatory factor 4 (IRF4) to increase MM cell adhesion to bone marrow stromal cells and accelerate MM cell homing to the bone marrow, which suggests KLF2 as a potential therapeutic target in MM [10]. Despite the possible mechanism of KIF2 in hematological malignancies, little is known about its clinical role in hematological malignancy patients. In the current study, we discovered that KLF2 could distinguish MM patients from health donors. Besides, it was positively correlated with ISS stage and R-ISS stage. Furthermore, its high expression was associated with decreased CR and worse PFS. These results might be explained by that (1) KIF2 might interact with several genes (such as AMPK, DT-13, KDM3A, and IRF4) to promote cell proliferation, subsequently increasing risk of MM occurrence [10, 29]. Thus, KLF2 could distinguish MM patients from health donors. (2) KLF2 might be related to abnormal albumin, β2-MG, or LDH via regulating various genes, and thereby associated with raised ISS stage and R-ISS stage in MM patients. (3) KLF2 might be associated with *t* (4; 16) (an important cytogenetic change of MM), and thereby related to increased R-ISS stage, eventually causing poor prognosis in MM patients.

Besides, we excluded these patients who died during induction therapy. The main reason was that most of them died due to other reasons such as infection but not the MM itself, and the inclusion of these patients in this study might influence the findings in this study; thus, we excluded them from the present study.

Some limitations still existed in the present study as follows: (1) a total of 72 de novo symptomatic MM patients were enrolled in this study, while there were just 30 healthy BM donors. This might be due to that it was hard to enroll enough BM donors for study use. (2) All patients were from our hospital. Further multicenter study is necessary. (3) The detailed mechanisms of EGFL7, ITGB3, and KLF2 underlying MM were unclear. Hence, more in vitro experiments are still needed. (4) The change of EGFL7, ITGB3, and KLF2 was not determined in this study, and further study is needed to highlight their prognostic value in MM. (5) The sample size was relatively small, and these findings needed to be verified in the further big-sample-size study.

In conclusion, EGFL7, ITGB3, and KLF2 are inter-correlated, and three of them are related to increased MM risk. Also, they positively correlate with prognostic risk stratification stages and prognosis in MM patients. Of note, ITGB3 appears to have a strong prognostic value in MM patients.

## Supplementary Information

Below is the link to the electronic supplementary material.

Supplementary file1 (DOCX 16 KB)

Supplementary file2 Fig. 1. Relationship of EGFL7, ITGB3 and KLF2 with the immunoglobulin subtype. Correlation of EGFL7 (A), ITGB3 (B) and KLF2 (C) with immunoglobulin subtype (including IgG, IgA, and others). EGFL7: epidermal growth factor like protein-7; ITGB3: integrin subunit beta 3; KLF2: Kruppel-like factor 2; IgG, immunoglobulin G; IgA, immunoglobulin A. (DOCX 96 KB)
