# Supplementary material for: Relation among EGFL7, ITGB3, and KLF2 and their clinical implication in multiple myeloma patients: a prospective study
Source: Ir J Med Sci. 2021 Oct 11;191(5):1995–2001. doi: 10.1007/s11845-021-02781-2 (PMC9492554; doi:10.1007/s11845-021-02781-2)
Supplement: Supplementary file 1 — Supplementary file1 (DOCX 16 KB) [file 11845_2021_2781_MOESM1_ESM.docx]

**Supplemental Table 1.** Multivariate Cox’s proportional hazards regression analysis for PFS and OS

| Items | *P* value | HR | 95% CI | |
| --- | --- | --- | --- | --- |
|  |  |  | Lower | Upper |
| **Cox’s regression analysis for PFS** |  |  |  |  |
| ITGB3 expression (high vs. low) | 0.014 | 2.419 | 1.197 | 4.887 |
| **Cox’s regression analysis for OS** |  |  |  |  |
| ITGB3 expression (high vs. low) | 0.043 | 4.091 | 1.047 | 15.978 |
| t (14; 16) (yes vs. no) | 0.009 | 11.736 | 1.865 | 73.851 |
| Bone lesion (yes vs. no) | 0.085 | 5.997 | 0.783 | 45.953 |

PFS, progression-free survival; OS, overall survival; HR, hazard ratio; CI, confidence interval; ITGB3, integrin subunit beta 3.
