# Supplementary material for: Relation among EGFL7, ITGB3, and KLF2 and their clinical implication in multiple myeloma patients: a prospective study
Source: Ir J Med Sci. 2021 Oct 11;191(5):1995–2001. doi: 10.1007/s11845-021-02781-2 (PMC9492554; doi:10.1007/s11845-021-02781-2)
Supplement: Supplementary file 2 — Supplementary file2 Fig. 1. Relationship of EGFL7, ITGB3 and KLF2 with the immunoglobulin subtype. Correlation of EGFL7 (A), ITGB3 (B) and KLF2 (C) with immunoglobulin subtype (including IgG, IgA, and others). EGFL7: epidermal growth factor like protein-7; ITGB3: integrin subunit beta 3; KLF2: Kruppel-like factor 2; IgG, immunoglobulin G; IgA, immunoglobulin A. (DOCX 96 KB) [file 11845_2021_2781_MOESM2_ESM.docx]

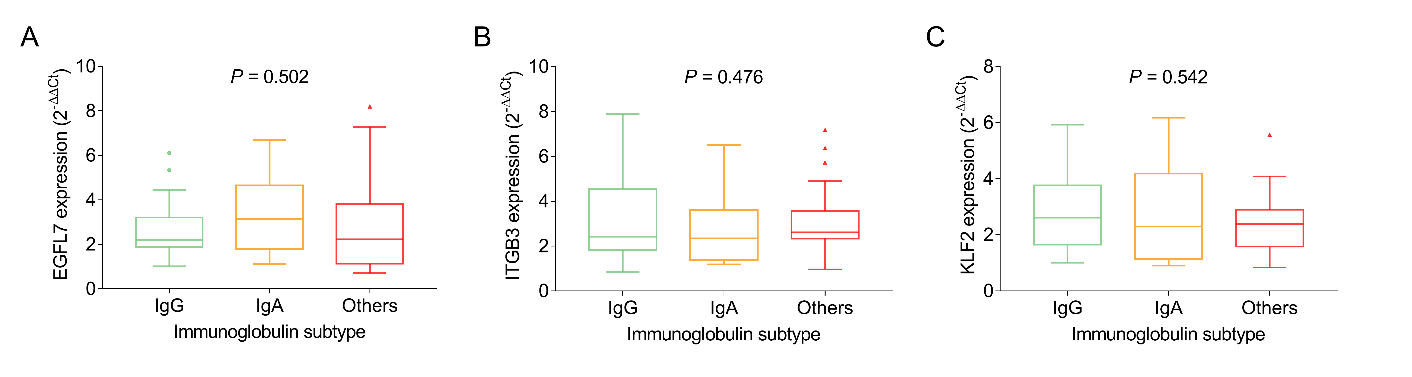


**Supplemental figure 1.** Relationship of EGFL7, ITGB3 and KLF2 with the

immunoglobulin subtype. Correlation of EGFL7 (**A**), ITGB3 (**B**) and KLF2 (**C**) with

immunoglobulin subtype (including IgG, IgA and others). EGFL7: epidermal growth

factor like protein-7; ITGB3: integrin subunit beta 3; KLF2: Kruppel-like factor 2; IgG,

immunoglobulin G; IgA, immunoglobulin A.
